# Multidirectional chromosome painting substantiates the occurrence of extensive genomic reshuffling within Accipitriformes

**DOI:** 10.1186/s12862-015-0484-0

**Published:** 2015-09-26

**Authors:** Wenhui Nie, Patricia C. M. O’Brien, Beiyuan Fu, Jinghuan Wang, Weiting Su, Kai He, Bertrand Bed’Hom, Vitaly Volobouev, Malcolm A. Ferguson-Smith, Gauthier Dobigny, Fengtang Yang

**Affiliations:** State Key Laboratory of Genetic Resources and Evolution, Kunming Institute of Zoology, Chinese Academy of Sciences, Kunming, Yunnan 650223 P R China; Cambridge Resource Centre for Comparative Genomics, Department of Veterinary Medicine, University of Cambridge, Cambridge, CB3 0ES UK; Wellcome Trust Sanger Institute, Wellcome Trust Genome Campus, Hinxton, Cambridge, CB10 1SA UK; INRA, AgroParisTech, UMR1313 Génétique Animale et Biologie Intégrative, Domaine de Vilvert-Bâtiment 320, 78352 Jouy-en-Josas Cedex, France; Muséum National d’Histoire Naturelle, Département Systématique et Evolution, UMR 7205 Origine, Structure et Evolution de la Biodiversité, 75005 Paris, France; Institut de Recherche pour le Développement, Centre de Biologie pour la Gestion des Populations (UMR IRD-INRA-Cirad-Montpellier SupAgro), Campus International de Baillarguet, CS30016, 34988 Montferrier-sur-Lez, France

**Keywords:** Accipitriformes, Fluorescent *in situ* hybridization, Multidirectional painting, Chromosomal rearrangements, Chromosome-based phylogenetics

## Abstract

**Background:**

Previous cross-species painting studies with probes from chicken (*Gallus gallus*) chromosomes 1–10 and a paint pool of nineteen microchromosomes have revealed that the drastic karyotypic reorganization in Accipitridae is due to extensive synteny disruptions and associations. However, the number of synteny association events and identities of microchromosomes involved in such synteny associations remain undefined, due to the lack of paint probes derived from individual chicken microchromosomes. Moreover, no genome-wide homology map between Accipitridae species and other avian species with atypical karyotype organization has been reported till now, and the karyotype evolution within Accipitriformes remains unclear.

**Results:**

To delineate the synteny-conserved segments in Accipitridae, a set of painting probes for the griffon vulture, *Gyps fulvus* (2n = 66) was generated from flow-sorted chromosomes. Together with previous generated probes from the stone curlew, *Burhinus oedicnemus* (2n = 42), a Charadriiformes species with atypical karyotype organization, we conducted multidirectional chromosome painting, including reciprocal chromosome painting between *B. oedicnemus* and *G. fulvus* and cross-species chromosome painting between *B. oedicnemus* and two accipitrid species (the Himalayan griffon, *G. himalayensis* 2n = 66, and the common buzzard, *Buteo buteo*, 2n = 68). In doing so, genome-wide homology maps between *B. oedicnemus* and three Accipitridae species were established. From there, a cladistic analysis using chromosomal characters and mapping of chromosomal changes on a consensus molecular phylogeny were conducted in order to search for cytogenetic signatures for different lineages within Accipitriformes.

**Conclusion:**

Our study confirmed that the genomes of the diurnal birds of prey, especially the genomes of species in Accipitriformes excluding Cathartidae, have been extensively reshuffled when compared to other bird lineages. The chromosomal rearrangements involved include both fusions and fissions. Our chromosome painting data indicated that the Palearctic common buzzard (BBU) shared several common chromosomal rearrangements with some Old World vultures, and was found to be more closely related to other Accipitridae than to Neotropical buteonine raptors from the karyotypic perspective. Using both a chromosome-based cladistic analysis as well as by mapping of chromosomal differences onto a molecular-based phylogenetic tree, we revealed a number of potential cytogenetic signatures that support the clade of Pandionidae (PHA) + Accipitridae. In addition, our cladistic analysis using chromosomal characters appears to support the placement of osprey (PHA) in Accipitridae.

**Electronic supplementary material:**

The online version of this article (doi:10.1186/s12862-015-0484-0) contains supplementary material, which is available to authorized users.

## Background

Most birds studied so far have highly conserved karyotypes, similar to that of the chicken (*Gallus gallus,* GGA, 2*n* = 78), consisting of several pairs of macrochromosomes and a mass of microchromosomes, with diploid chromosome number (2n) varying mainly from 76 to 84 [1, 2]. In contrast, the diurnal birds of prey have a unique karyotype organization that differs widely from the usually conserved genomic structure found in other bird lineages [3, 4]. In particular, Accipitridae species display the most atypical karyotypes known in Aves [5]. The more than fifty accipitrid species studied so far (reviewed in [6]) share the following karyotypic characteristics: 1) most species have diploid chromosome numbers varying from 66 to 68; 2) they lack large macrochromosomes; and 3) they have many medium-to small-sized bi-armed chromosomes and usually three to six pairs of microchromosomes [3, 5]. Only one pair of microchromosomes was found in the black-winged kite (*Elanus caeruleus*, 2*n* = 68) [7]. Previous karyotypic comparisons have suggested that such atypical karyotypes of accipitrids could have evolved from a typical bird karyotype by a series of fissions of macrochromosomes and translocations of some macrochromosome segments onto the microchromosomes and small acrocentrics [3]. However, the exact nature of chromosome structural rearrangements that took place in the karyotype evolution of the accipitrid lineage remains largely unclear.

Cross-species chromosome painting in birds, mostly with painting probes derived from flow-sorted GGA macrochromosomes, has allowed the establishment of reliable chromosome homologies between GGA and more than fifty avian species belonging to twelve orders (reviewed in [2], and [8–18]). To date, comparative chromosome painting with GGA chromosome-specific probes has been applied to nine species in Accipitridae: the Harpy eagle (*Harpia harpyja*, 2n = 58) [19], three Old World vultures (*Gypaetus barbatus*, 2*n* = 60; *Gyps rueppellii*, 2*n* = 66; *G. fulvus*, 2*n* = 66) [20], the white hawk [*Pseudastur albicollis* (= *Leucopternis albicollis*), 2*n* = 66] [9], the Japanese mountain hawk-eagle (*Nisaetus nipalensis orientalis*, 2*n* = 66) [13], and three species of Buteoninae [*Rupornis magnirostris*, *Buteo nitidus* (= *Asturina nitida*), *Buteogallus meridionalis*, 2*n* = 68] [16]. GGA 1–5 probes each detected two or more homologous chromosomes or chromosome segments in the karyotypes of accipitrid species studied so far, demonstrating that synteny disruption exists in the GGA 1–5 homologues. GGA 6–10 probes each revealed one pair of homologous chromosomes or chromosome segments, indicating that the GGA 6–10 homologues are conserved in Accipitridae. Besides GGA 1–10 probes, several sets of probes derived from GGA microchromosome pools were also used in a few avian chromosome painting studies [12–14, 19, 21, 22]. A paint pool for nineteen GGA microchromosomes detected homology with a lesser number of chromosomal segments and smaller chromosomes in two accipitrid species (the Harpy eagle, *H. harpyja* and the Japanese mountain hawk-eagle, *N. nipalensis orientalis*), suggesting that the decrease in chromosome number in Accipitridae was due to fusions between microchromosomes and fusions of microchromosomes with larger chromosomes [13, 19]. However, the GGA microchromosomes homologues involved in the fusions remain unidentified due to the use of a paint pool containing multiple undefined GGA microchromosomes. To better characterize the microchromosomes involved in rearrangements as well as the evolutionary breakpoints along GGA macrochromosomes, chromosome-specific probes from more bird species, in particular those with atypical karyotypes, are required.

Most recently, two complete sets of painting probes have been generated from two neoavian species: the stone curlew (*Burhinus oedicnemus*, BOE, 2*n* = 42, Charadriiformes) [14] and the white hawk (*P. albicollis*, PAL, 2n = 66, Accipitriformes) [9]. BOE has one of the lowest diploid numbers reported so far in birds. Using the GGA and BOE chromosome painting probes, the first reciprocal chromosome painting between avian species was performed, and a comparative chromosome map between BOE and GGA has been established [14]. The whole set of BOE painting probes was subsequently used to delineate chromosome homology between BOE and representatives of five avian orders [23]. In addition, PAL probes were used to detect chromosomal homologies between GGA 1–10 and that of PAL [9], between PAL and three macrochromosomes of a New World vulture, the turkey vulture (*Cathartes aura*, 2n = 80) [15], three Buteoninae species (2n = 68) [16] and two Turdidae songbirds (2n = 78, Passeriformes) [17]. Up to now, all cross-species chromosome painting studies in birds have involved either two species with typical avian karyotypes, or one species with a typical and the other with an atypical karyotype. No genome-wide comparative chromosome painting between two bird species with atypical karyotypes has ever been performed.

In the past, the diurnal birds of prey constituted the Order Falconiformes and were classified into five families (i.e. Cathartidae, Falconidae, Accipitridae, and the two monotypic families Pandionidae and Sagittariidae) [24]. However, the status of Falconiformes as a natural (i.e. monophyletic) group was long under debate due to marked heterogeneity in karyological, morphological and mitochondrial (mt) gene order found in different families of Falconiformes (reviewed in [25]). The most recent molecular phylogeny studies retrieved strong support for a clade containing the secretary bird (Sagittariidae), the osprey (Pandionidae) and the traditional accipitrids (e.g., [26–33]). Consequently, Falconiformes now only includes the family Falconidae, while the other diurnal birds of prey constitute a new order, Accipitriformes. Here, we adopted this new taxonomic classification for the diurnal birds of prey. In Accipitriformes, there are some important unresolved issues concerning the rank of the osprey and the status of the Old World vultures as well as the Buteoninae as monophyletic or polyphyletic groups. The osprey was often recognized as a family-level taxa (e.g. [26–29, 32]); however, in some studies, together with the secretary bird, the osprey was assigned the rank of subfamily within Accipitridae (e.g. [30]). Recent molecular studies suggested that the Old World vultures and the Buteoninae were polyphyletic [26, 27, 29, 31, 32]. Up to now, no Sagittariidae species (i.e. the secretary bird) has ever been studied using chromosome painting. Besides Accipitridae, three species from other families of Accipitriformes and three species from Falconiformes have been studied by this technique [12, 15, 18, 34]. The availability of chromosome painting data from different diurnal birds of prey families offers us the opportunity to decipher the genomic rearrangements within the diurnal birds of prey and to search for cytogenetic evidence for the phylogenetic relationships within the Accipitriformes.

In this context, we here report the generation of 1) a complete set of painting probes that cover the whole genome of the griffon vulture (*G. fulvus*, GFU, 2n = 66), an Old World vulture, through bivariate flow sorting and DOP-PCR amplification; and 2) comparative chromosome maps based on reciprocal chromosome painting between BOE and GFU, two species belonging to different orders with atypical karyotypes (namely, Charadriiformes and Accipitriformes). Moreover, the whole set of BOE probes was hybridized to metaphase chromosomes of two species in Accipitridae, Himalayan griffon (*G. himalayensis*, GHI, 2n = 66) and common buzzard (*Buteo buteo*, BBU, 2n = 68) to detect karyotypic differences between these species. A cladistic analysis using chromosomal rearrangements as well as the mapping of ZOO-FISH-inferred chromosomal differences on a concensus molecular phylogeny were performed, providing further insight into the origin and evolution of the extensively rearranged Accipitriformes genome.

## Results

### *Characterization of the* G. fulvus *flow karyotype*

**Fig. 1:**
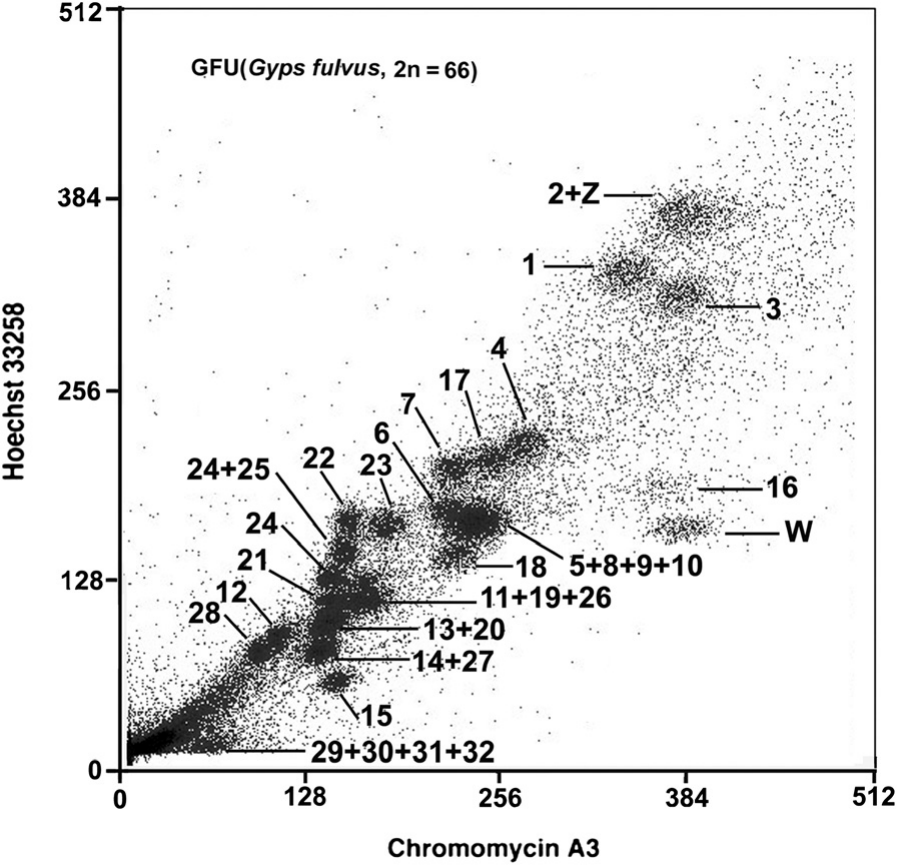
Bivariate flow karyotype of *G. fulvus* (2n = 66) with chromosome assignments

As reported previously [3], GFU has a 2n = 66 karyotype, including fifteen meta-or sub-metacentric chromosomes, thirteen acrocentric chromosomes, four pairs of dot-like microchromosomes, together with a large submetacentric Z and a medium-sized submetacentric W. The thirty-three pairs of GFU chromosomes were resolved into twenty-three flow peaks (Fig. 1). Hybridizing paints prepared from individual peaks onto GFU metaphases established the identity of the chromosomes contained in each peak. Reciprocal chromosome painting between GFU and BOE was also performed to resolve any ambiguity in the chromosomal assignment of each flow peak and, in particular, of the peaks that contained multiple chromosomes (see below). The majority of flow peaks each contained only one type of GFU chromosome, with the exception of seven peaks, of which four peaks each contained two GFU chromosomes (GFU 2 + Z, 13 + 20, 14 + 27 and 24 + 25), one peak contained three GFU chromosomes (GFU 11 + 19 + 26) and the remaining two peaks each contained four GFU chromosomes (GFU 5 + 8 + 9 + 10 and 29 + 30 + 31 + 32). Moreover, the two homologues of chromosome 24 were each sorted into a different flow peak. Thus, a set of chromosome painting probes that cover the entire genome of GFU was generated.

### *Reciprocal chromosome painting between* G. fulvus *and* B. oedicnemus

**Fig. 2:**
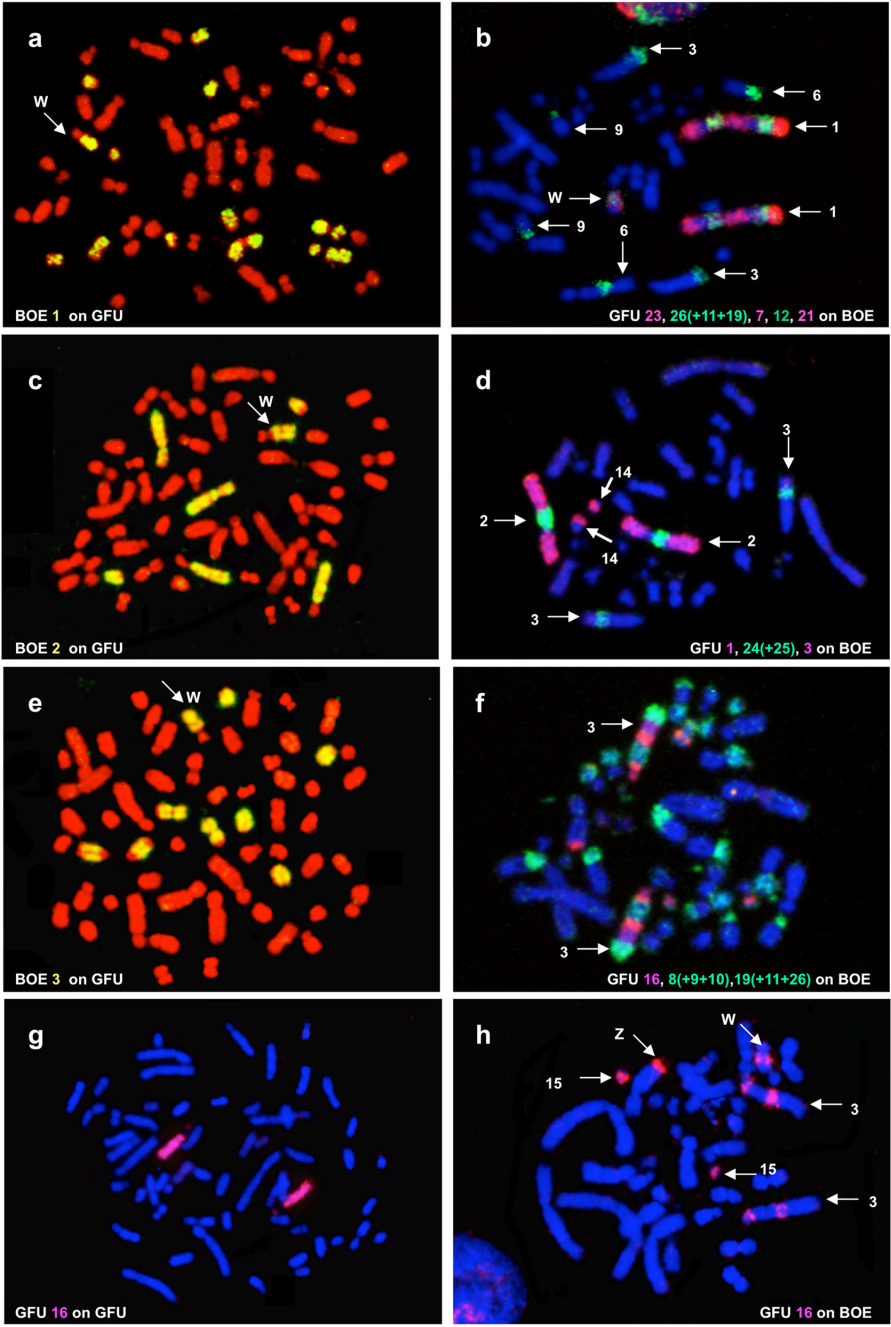
Reciprocal chromosome painting between BOE and GFU. **a** BOE 1 probe hybridized to seven pairs of GFU chromosomes. **b** Probes from GFU 23 (red), 26 (+11 + 19) (green), 7 (red), 22 (green) and 21 (red) painted BOE 1. **c** BOE 2 probe hybridized to three pairs of GFU chromosomes. **d** Probes from GFU 1 (red), 24 (+25) (green) and 3 (red) labeled BOE 2. **e** BOE 3 probe hybridized to four pairs of GFU chromosomes. **f** Probes from GFU 16 (red), 8 (+5 + 9 + 10) (green) and 19 (green) probes painted BOE 3. **g** GFU 16 probe labeled GFU16. **h** GFU 16 probe hybridized to BOE 3 and 15. Note that cross-hybridization signals were also detected on BOE Z and W. Due to the fact that one of the used GFU probes in (**b**), (**d**) and (**f**) contained two or more GFU chromosomes or that one used GFU probe gave signals on two or more BOE chromosomes, besides BOE 1–3, other BOE chromosomes also were painted in (**b**), (**d**) and (**f**)

**Fig. 3:**
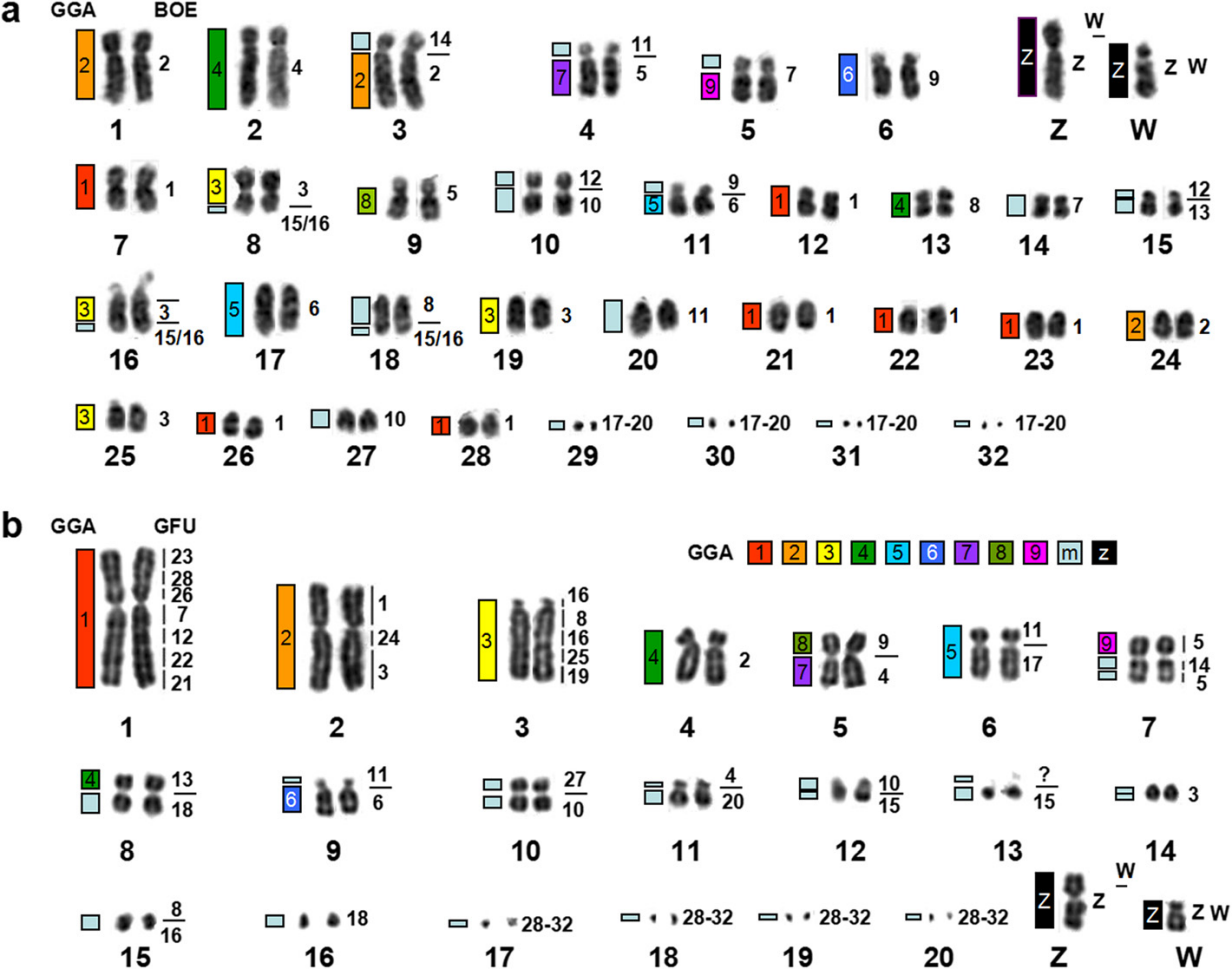
**a** DAPI-banded karyotype of GFU with the assignment of homologies to BOE. **b** DAPI-banded karyotype of BOE with the assignment of homologies to GFU on the right. Homologies to GGA are indicated on the left of each GFU and GHI and BBU chromosome

Together with BOE [14] and PAL [9], the set of GFU painting probes represents the third set of paints that have been generated from Neoaves. Reciprocal chromosome painting between GFU and BOE established chromosome homologies between these species and defined chromosomes contained in GFU flow peaks. FISH examples are shown in Fig. 2, and results of reciprocal chromosome painting between BOE and GFU are summarized onto DAPI–banded karyotypes of GFU (Fig. 3a) and BOE (Fig. 3b), respectively.

**Fig. 4:**
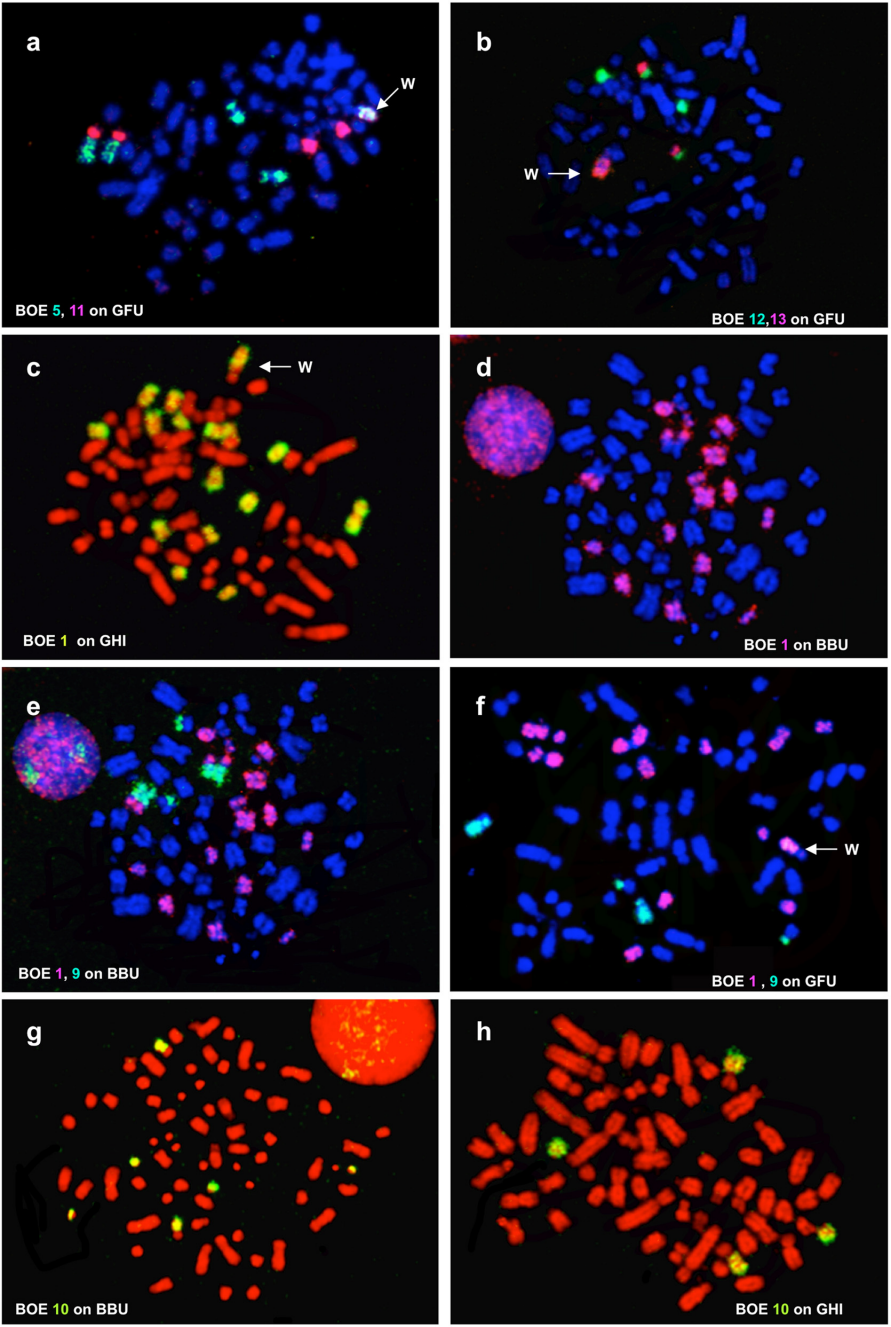
Examples of cross-species chromosome painting with BOE painting probes. **a** Hybridization of BOE 5 (green) and 11 (red) probes to GFU chromosomes. **b** Hybridization of BOE 12 (green) and 13 (red) probes to GFU chromosomes. **c** Hybridization of BOE 1 probe to seven pairs of GHI chromosomes. **d** Hybridization of BOE 1 probe to eight pairs of BBU chromosomes. **e** Hybridization of BOE 1 (red) and 9 (green) probes to BBU chromosomes. **f** Hybridization of BOE 1 (red) and 9 (green) probes to GFU chromosomes. **g** Hybridization of BOE 10 probe to three pairs of BBU chromosomes. **h** Hybridization of BOE 10 probe to two pairs of GHI chromosomes. Note that cross-hybridization signal was also detected on the W chromosomes of GFU (**a**, **b**, **f**) and GHI (**c**)

Probes derived from BOE 1–3 (= GGA 1–3) detected seven (Fig. 2a), three (Fig. 2c) and four (Fig. 2e) homologous segments in GFU genome, respectively, in addition to cross-hybridization signals on the long arm of GFU W (Fig. 2a, c and e). BOE 5 (= GGA 8 + 7)-12 (= two GGA microchromosomes) probes each hybridized to two different GFU chromosomes or chromosome segments (Fig. 4a, b and f). BOE 4 (= GGA 4q), 13 (= two GGA microchromosomes) and 14 (= two GGA microchromosomes) probes each painted one GFU chromosome or chromosome segment (Fig. 4b). BOE 15 + 16 (= two GGA microchromosomes) probe gave signals on three pairs of GFU chromosomes. A paint derived from four microchromosomes (i.e., BOE 17 + 18 + 19 + 20) hybridized to the four pairs of GFU microchromosomes (29, 30, 31 and 32). In total, BOE autosomal probes revealed forty homologous chromosomal segments in the GFU genome (Fig. 3a).

GFU autosomal probes revealed forty-one segments of conserved synteny in the BOE genome (Fig. 3b). Twenty-four GFU chromosomes (1, 2, 6, 7, 9, 12–14 and 17–32) each corresponded to one BOE chromosome or chromosome segment; eight GFU chromosomes (3–5, 8, 10, 11, 15 and 16) each corresponded to two BOE chromosomes or chromosome segments. In agreement with the finding that BOE 1–3 (= GGA 1–3) probes gave signals on multiple GFU chromosomes, BOE 1–3 (= GGA 1–3) were each painted by three or more GFU probes (Fig. 2b, d and f). The GFU 16 probe (Fig. 2g) gave two discrete signals on BOE 3 and one signal on BOE 15 (Fig. 2h). Cross-hybridization signals were also found on BOE Z and W chromosomes (Fig. 2h). Finally, the pooled probe from the four smallest GFU microchromosomes (29–32) painted the smallest four pairs of BOE microchromosomes (17–20).

### *Hybridizing* B. oedicnemus *probes onto metaphases of* G. himalayensis *and* B. buteo

**Fig. 5:**
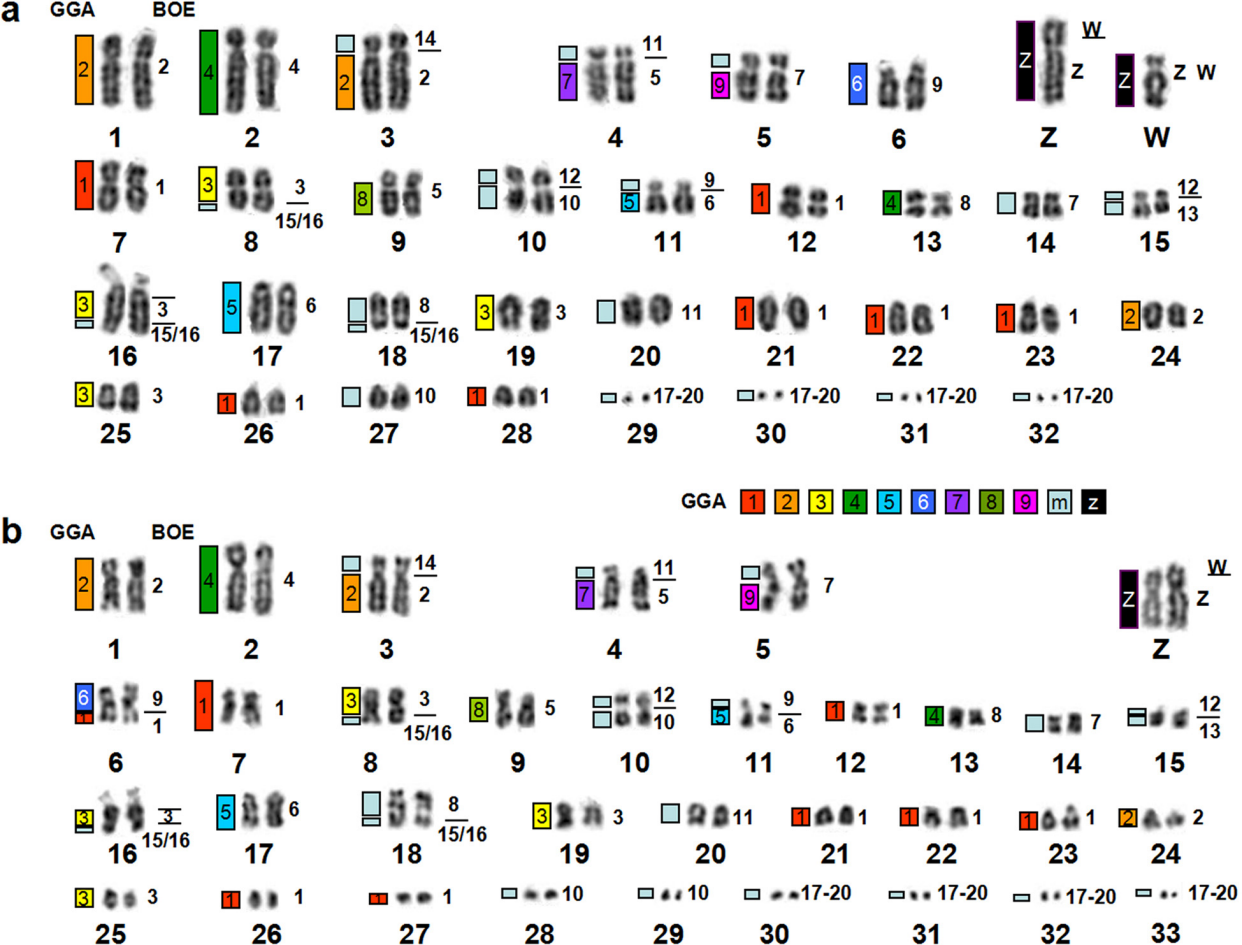
**a** DAPI-banded karyotype of GHI with assignment of homologies to BOE. **b** DAPI-banded karyotype of BBU with assignment of homologies to BOE. Homologies to GGA are indicated on the left of each BOE chromosome

Like other *Gyps* species, *G. himalayensis* (GHI) has a 2n = 66 karyotype, which is similar to that of GFU. As expected, the patterns produced by hybridizing each BOE probe to GFU and GHI chromosomes were identical (Fig. 3a and Fig. 5a). Examples of BOE probes hybridizing to chromosomes of GHI are shown in Fig. 4c and h. Accordingly, paints from BOE autosomes detected forty homologous segments in GHI (Fig. 5a).

The male *B. buteo* (BBU) shows a 2n = 68 karyotype, comprising fifteen pairs of meta-or sub-metacentric chromosomes, fourteen pairs of acrocentric chromosomes, four pairs of microchromosomes and one pair of large submetacentric Z chromosomes (Fig. 5b), as previously reported [5]. Painting probes from BOE were hybridized to metaphase spreads of BBU. FISH examples are shown in Fig. 4d, e and g. Results are summarized using a DAPI-banded karyotype of BBU (Fig. 5b). Hybridization patterns produced by each BOE probe were very similar in GFU, GHI and BBU. Only two BOE probes gave different numbers of signals in BBU, GFU and GHI. BOE 1 (= GGA 1) probe painted eight pairs of BBU chromosomes (Fig. 4d and e), but only seven pairs of chromosomes in GFU and GHI (Figs. 2a and 4c, f); BOE 10 (= GGA 10 + 1 microchromosome) probe hybridized onto three pairs of BBU chromosomes (Fig. 4g) but two pairs of chromosomes in GFU and GHI (Fig. 4h). In total, probes from BOE autosomes revealed forty-two homologous segments in the BBU genome.

To facilitate comparison of homologous chromosomal segments across bird species, the homologous GGA chromosome segments as inferred from the GGA–BOE comparative chromosomal map [14] were also indicated to the left of GFU, BOE, GHI and BBU chromosomes (Figs. 3 and 5).

### Chromosomal phylogeny of the diurnal birds of prey

**Fig. 6:**
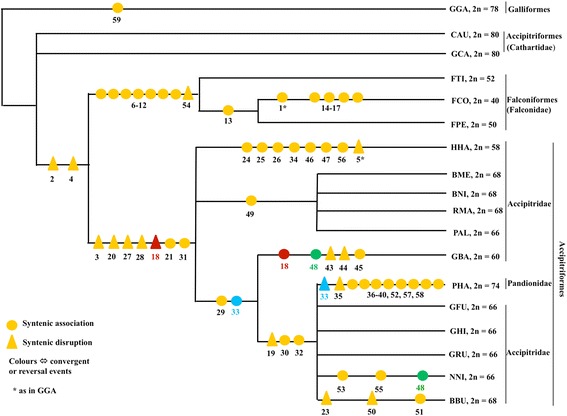
Chromosomal phylogeny generated by PAUP, with chromosomal rearrangements as *a posteriori* polarized characters. Among 59 chromosome changes, 38 corresponded to syntenic associations, 15 were syntenic disruptions and 3 represented convergent or reversal events. Characters 22, 41 and 42 were not mapped onto the tree due to the ambiguities regarding their interpretation (see text for details). Numbers on the tree stand for chromosome characters that are described in Table 1. Published chromosome painting data for Falconidae (Nishida et al., [12]), Pandionidae (Nishida et al., [18]), Accipitridae (de Oliveira et al., [10, 16, 19]; Nanda et al., [20]; Nishida et al., [13]), Cathartidae (Raudsepp et al., [34]; Tagliarini et al., [15]) and the data in this study were used for this figure. BBU, *Buteo buteo*; BME, *Buteogallus meridionalis*; BNI, *Buteo nitida* (= *Asturina nitida*); CAU, *Cathartes aura*; FCO, *Falco columbarius*; FPE, *Falco peregrinus*; FTI, *Falco tinnunculus*; GBA, *Gypaetus barbatus*; GCA, *Gymnogyps californianus*; GFU, *Gyps fulvus*; GHI, *Gyps himalayensis*; GRU, *Gyps rueppellii*; HHA, *Harpia harpyja*; NNI, *Nisaetus nipalensis orientialis*; PAL, *Pseudastur albicollis* (= *Leucopternis albicollis*); PHA, *Pandion haliaetus*; RMA, *Rupornis magnirostris*

In total, 59 chromosomal characters were constructed from available inter-species chromosome painting data including 14 species of Accipitriformes and 3 species of Falconiformes (Table 1). The exhaustive search allowed us to retrieve 10 equally most parsimonious trees (L = 67; Additional file 1: Figure S1). These 10 trees were largely congruent in showing three main clades: one represented the New World vultures; one grouped all falconid representatives, while the other gathered all accipitrid species. Furthermore, the grouping of Neotropical Buteoninae (BNI, BME, RMA, PAL) was systematically retrieved while the Palearctic buteonine species BBU was consistently found closer to an assemblage made of accipitrid (GFU and NNI) as well as pandionid (PHA) species. The main topologic instability originated from the uncertainty in the branching of GBA and HHA (see all 10 most parsimonious trees in Additional file 1: Figure S1). The 50 % majority-rule tree is represented in Fig. 6. *A posteriori* polarization of character changes along this tree using the outgroup comparison criterion showed that more than 50 chromosomal rearrangements occurred during the karyotypic evolution of the diurnal birds of prey studied here (Fig. 6). Among them, 53 could be unambiguously characterized: 38 corresponded to syntenic associations while 15 were syntenic disruptions (Fig. 6). Three characters (characters 18, 33 and 48) could represent convergent or reversal events. Three other characters were hardly interpretable due to the topologic instability of GBA and/or HHA (characters 22, 41 and 42).

**Table 1:** Data matrix used in the PAUP analysis

Character no.	Character (GGA)	GGA (2*n* = 78)	Falconiformes			Accipitriformes								
						Cathartidae	Pandiondae	Accipitridae						
			FTI (2*n* = 52)	FPE (2*n* = 50)	FCO (2*n* = 40)	GCA CAU (2*n* = 80)	PHA (2*n* = 74)	GBA (2*n* = 60)	GFU GHI GRU (2*n* = 66)	HHA (2*n* = 58)	NNI (2*n* = 66)	BNI BME RMA (2*n* = 68)	PAL (2*n* = 66)	BBU (2*n* = 68)
1	1p/1q	0	1	1	0	0	1	1	1	1	1	1	1	1
2	2p/2q	0	1	1	1	0	1	1	1	1	1	1	1	1
3	3qprx/3qmed	0	0	0	0	0	1	1	1	1	1	1	1	1
4	5qprx/5qdis	0	1	1	1	0	1	1	1	1	1	1	1	1
5	5qprx/mic1	0	1	1	1	0	1	1	1	0	1	1	1	1
6	2p/mic2	0	1	1	1	0	0	0	0	0	0	0	0	0
7	2q/mic3	0	1	1	1	0	0	0	0	0	0	0	0	0
8	4p/mic4	0	1	1	1	0	0	0	0	0	0	0	0	0
9	4q/mic5	0	1	1	1	0	0	0	0	0	0	0	0	0
10	5qmed/mic6	0	1	1	1	0	0	0	0	0	0	0	0	0
11	6/mic7	0	1	1	1	0	0	0	0	0	0	0	0	0
12	7/mic8	0	1	1	1	0	0	0	0	0	0	0	0	0
13	(5qdis + mic6)/(7 + mic8)	0	0	1	1	0	0	0	0	0	0	0	0	0
14	3qdis-1/(4q + mic5)	0	0	0	1	0	0	0	0	0	0	0	0	0
15	3qprx-1/(2p + mic2)	0	0	0	1	0	0	0	0	0	0	0	0	0
16	(2q + mic3)/(5qprx + mic1)	0	0	0	1	0	0	0	0	0	0	0	0	0
17	8/(6 + mic7)	0	0	0	1	0	0	0	0	0	0	0	0	0
18	1pdis/1pmed	0	0	0	0	0	1	0	1	1	1	1	1	1
19	1pmed/1pprx	0	0	0	0	0	1	0	1	0	1	0	0	1
20	1qprx/1qmed	0	0	0	0	0	1	1	1	1	1	1	1	1
21	1qmed/1qdis	0	0	0	0	0	1	1	1	1	1	1	1	1
22	1qdis-a/1qdis-b	0	0	0	0	0	1	0	1	1	1	0	0	1
23	1pdis-a/1pdis-b	0	0	0	0	0	0	0	0	0	0	0	0	1
24	1seg-n1/mic9	0	0	0	0	0	0	0	0	1	0	0	0	0
25	1seg-n2/mic10	0	0	0	0	0	0	0	0	1	0	0	0	0
26	(1seg-n1 + mic9)/(1seg-n2 + mic10)	0	0	0	0	0	0	0	0	1	0	0	0	0
27	3qmed-1/3qmed-2	0	0	0	0	0	1	1	1	1	1	1	1	1
28	3qmed-2/3qdis	0	0	0	0	0	1	1	1	1	1	1	1	1
29	3qprx/mic11	0	0	0	0	0	1	1	1	0	1	0	0	1
30	3qmed-1/mic12	0	0	0	0	0	1	0	1	0	1	0	0	1
31	7/mic13	0	0	0	0	0	1	1	1	1	1	1	1	1
32	8/mic14	0	0	0	0	0	1	0	1	0	1	0	0	1
33	2qprx/mic15	0	0	0	0	0	0	1	1	0	1	0	0	1
34	2p/mic16	0	0	0	0	0	0	0	0	1	0	0	0	0
35	6qprx/6qdis	0	0	0	0	0	1	0	0	0	0	0	0	0
36	1seg-na/9	0	0	0	0	0	1	0	0	0	0	0	0	0
37	1seg-nb/4p	0	0	0	0	0	1	0	0	0	0	0	0	0
38	1seg-nc/6qprx	0	0	0	0	0	1	0	0	0	0	0	0	0
39	1seg-nd/mic16	0	0	0	0	0	1	0	0	0	0	0	0	0
40	1seg-ne/mic17	0	0	0	0	0	1	0	0	0	0	0	0	0
41	9/mic18	0	0	0	0	0	0	1	1	1	1	0	0	1
42	2qprx/2qdis	0	0	0	0	0	0	1	1	0	1	1	1	1
43	2qdis-a/2qdis-b	0	0	0	0	0	0	1	0	0	0	0	0	0
44	1seg-nA/1seg-nB	0	0	0	0	0	0	1	0	0	0	0	0	0
45	1seg-nC/3qmed-1	0	0	0	0	0	0	1	0	0	0	0	0	0
46	6/mic-NOR	0	0	0	0	0	0	0	0	1	0	0	0	0
47	(3q-n + mic19)/5 dis	0	0	0	0	0	0	0	0	1	0	0	0	0
48	6/mic20	0	0	0	0	0	0	1	0	0	1	0	0	0
49	1pdis/6	0	0	0	0	0	0	0	0	0	0	1	1	0
50	mic21-a/mic21-b	0	0	0	0	0	0	0	0	0	0	0	0	1
51	1pdis-2/6	0	0	0	0	0	0	0	0	0	0	0	0	1
52	2p/mic22	0	0	0	0	0	1	0	0	0	0	0	0	0
53	2qprx/mic23	0	0	0	0	0	0	0	0	0	1	0	0	0
54	3qprx-1/3qdis-1	0	1	1	1	0	0	0	0	0	0	0	0	0
55	3qmed-2/mic24	0	0	0	0	0	0	0	0	0	1	0	0	0
56	3q-n/mic25	0	0	0	0	0	0	0	0	1	0	0	0	0
57	5qdis/mic26	0	0	0	0	0	1	0	0	0	0	0	0	0
58	6qdis/mic27	0	0	0	0	0	1	0	0	0	0	0	0	0
59	4p/4q	1	0	0	0	0	0	0	0	0	0	0	0	0

BNI, *Buteo nitida* (= *Asturina nitida*); BBU, *Buteo buteo*; BME, *Buteogallus meridionalis*; CAU, *Cathartes aura*; FCO, *Falco columbarius*; FPE, *Falco peregrinus*; FTI, *Falco tinnunculus*; GBA, *Gypaetus barbatus*; GCA, *Gymnogyps californianus*; GFU, *Gyps fulvus*; GHI, *Gyps himalayensis*; GRU, *Gyps rueppellii*; HHA, *Harpia harpyja*; PAL, *Pseudastur albicollis* (= *Leucopternis albicollis*); NNI, *Nisaetus nipalensis orientialis*; PHA, *Pandion haliaetus*; RMA, *Rupornis magnirostris*

Note: Chromosomal rearrangement characters used in this table were from previous published data and current study (see the section of materials for details). In the column of “Character (GGA)”, the individual numbers or the numbers before alphabets represent the numbers of homologous GGA chromosomes or chromosomal segments. “p”: the short arm of a given chromosome; “q”: the long arm of a given chromosome; “seg”: a segment on a given chromosome; “prx”: the part which is near to the centromere of a given chromosome or its arm; “med”: the part which is in the medial of a given chromosome or its arm; “dis”: the part which is distal to the centromere of a given chromosome or its arm; “mic”: the homologous GGA microchromosome. The chromosomal rearrangements in the diurnal birds of prey involved many homologous GGA microchromosomes. However, it is impossible to identify each homologous GGA microchromosomes due to the lack of single GGA microchromosome-specific probes. The numbers after “mic” are arbitrary numbers assigned for GGA microchromosomes involved in different species. GGA 1 is homologous to 4–8 pairs of chromosomes in different Accipitriformes species. Whereby the homologues of GGA 1 segments involved in chromosome arrangements could not be identified unambiguously based chromosome banding and painting data, different symbols were used to represent the homologues of GGA 1 segments in different species, such as 1 seg-n1 and 1seg-n2 in HHA, 1seg-nA, 1seg-nB and 1seg-nC in GBA, and 1seg-na to 1seg-ne in PHA

As an alternative approach to the above-mentioned parsimony-based cladistic analysis. these chromosomal characters were also mapped onto a consensual molecular tree generated by combining recent phylogenetic data in the literature [29, 32, 33] (Additional file 2: Figure S2). This treatment allowed the visualization of potential cytogenetic signature rearrangement(s) that could characterize the major lineages observed on the molecular-based consensus phylogeny. In congruence with the cladistic analysis, such an approach shows that different diurnal birds of prey taxa underwent different chromosomal changes, although many chromosomal characters were inferred to appear independently in different lineages. This is an indication of either true homoplasy or potential uncertainty in the branching as suggested by the molecular topology.

## Discussion

The generation of probes from GFU, a species of the Old World vultures, the application of both BOE and GFU probes to comparative painting in more accipitrid representatives as well as a cladistics analysis permitting proper *a posteriori* polarization of evolutionary events have enabled us to define further the types and number of chromosomal rearrangements that have occurred during the karyotypic diversification of Falconiformes and Accipitriforms, with special emphasis on Falconidae, Accipitridae and Pandionidae.

Our painting results with BOE probes have demonstrated that the two 2n = 66 Old World vultures (GFU and GHI) have identical karyotypes (Figs. 3a and 5a), and that at least twelve syntenic disruptions and nine segmental associations differentiate the karyotype of BOE from those of GFU and GHI. In a previous study, the hybridization patterns produced by GGA 1–10 probes were shown to be identical also between GFU and GRU (*G. rueppellii*) [20]. These results indicated that extant *Gyps* vultures have undergone no interchromosomal rearrangements during their divergence.

**Fig. 7:**
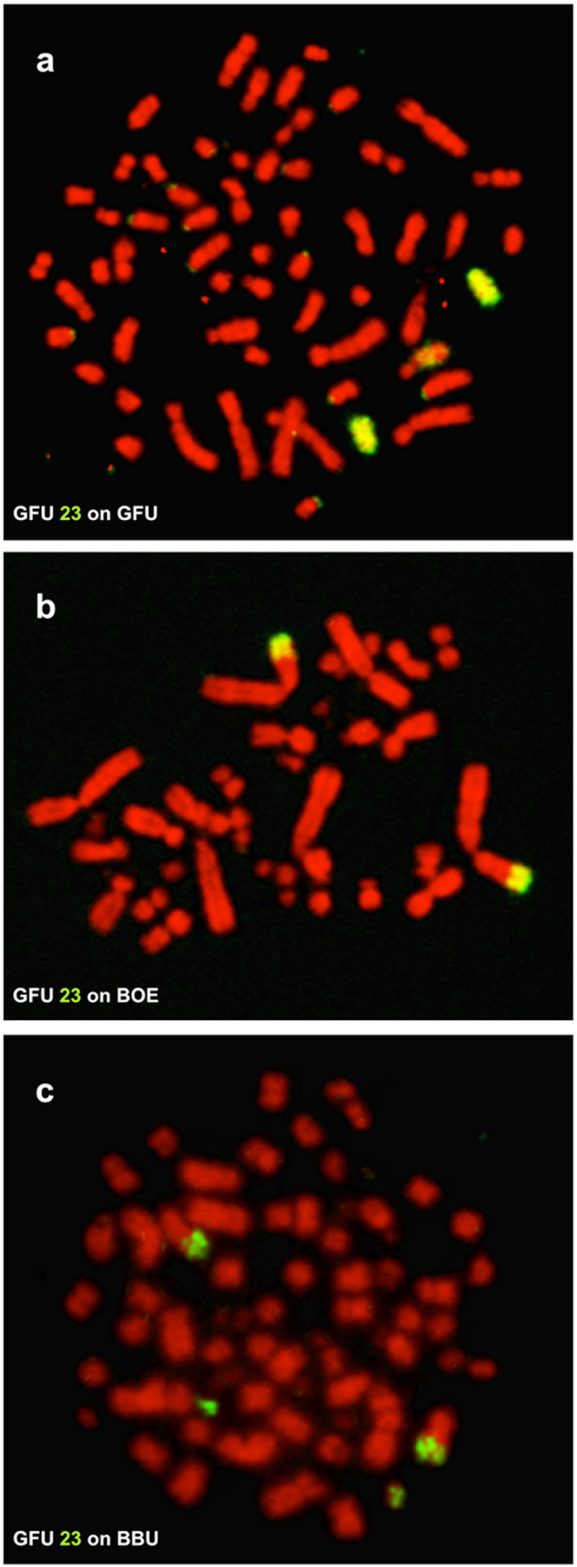
Examples of cross-species chromosome painting with GFU probes. **a** Hybridization of GFU 23 probe to GFU chromosomes. **b** Hybridization of GFU 23 probe to BOE 1pter. **c** Hybridization of GFU 23 probe to BBU chromosomes

The hybridization patterns of BOE probes in BBU (Fig. 5b) were very similar to those observed in GFU and GHI. Fourteen syntenic disruptions and ten syntenic associations were revealed in the karyotype of BBU by BOE probes. Among these rearrangements, eleven disruptions (characters 1–4, 18–22, 27 and 28) and eight associations (characters 5, 29–33, 41 and 42) detected in GFU and GHI by BOE probes were also shared with BBU. The karyotype differences between BBU (2n = 68) and the 2n = 66 Old World vultures (GFU and GHI) could have resulted from two further syntenic disruptions of homologous segments of BOE 1 and 10 (= GGA 1 and one GGA microchromosome, characters 23 and 50) and one further syntenic association (BOE 1 + 9 = GGA 1 + 6, character 51). Since multiple chromosomes in BBU, GFU and GHI show homologies with BOE 1 (= GGA 1), it is difficult to define which homologous segment of BOE 1 has undergone further fission in BBU based only on painting results from BOE probes. Further hybridization with GFU probes onto chromosomes of GFU, BOE and BBU allowed us to address this uncertainty (Fig. 7). The results indicate that the GFU 23 (Fig. 7a) homologous segment (= BOE 1pter = GGA 1pter, Fig. 7b) has undergone a syntenic disruption in BBU (Fig. 7c). Therefore, further syntenic disruptions of an ancestral macrochromosome segment (= GGA 1pter, character 23) and a microchromosome (= one segment of BOE 10 = one GGA microchromosome, character 50) could have occurred during chromosome evolution in BBU. Moreover, the complete conservation of BOE 17–20 and GFU 29–30 in multiple species studied here and in the rock pigeon (*Columba livia*, 2n = 80, Columbiformes) as revealed by BOE 17–20 probes [20] suggests that these four pairs of dot-shaped microchromosomes have a much more ancient origin, most likely already being present in the ancestral bird karyotype.

Previous comparative cytogenetic analyses indicated that the variation in karyotypes in the diurnal birds of prey was much wider than that in other avian groups, each family having its distinct karyotype features [3]. To date, including the current study, chromosome painting has been performed in fourteen species belonging to three families of the Accipitriformes and three species of Falconiformes. Only the monotypic family Sagittaridae, represented by the Secretary bird (*Sagittarius serpentarius*), has not been studied yet by chromosome painting. Table 2 lists the correspondence between GGA 1–5 and their counterparts in BOE and seventeen species belonging to Accipitriformes and Falconiformes. These homology data, together with chromosome painting data on other birds, allowed us to deduce the possible process of karyotype evolution in Accipitriformes through a proper cladistics analysis and mapping the chromosomal changes onto a consensus molecular tree.

**Table 2:** Homologies between GGA 1–5 and chromosomes of BOE and seventeen species in Accipitriformes and Falconiformes revealed by chromosome painting

Species	2n	Homologues of chicken 1-5					References
		GGA1	GGA2	GGA3	GGA4	GGA5	
Charadriiformes							
*Burhinus oedicnemus*, BOE	42	1	2	3	4, 8p	6	Nie et al., [14]
Accipitriformes							
Cathartidae							
*Gymnogyps californianus*, GCA	80	1	2	3	4, 9	5	Raudsepp et al., [34]
*Cathartes aura*, CAU	80	1	2	3	4, 9	5	Tagliarini et al., [15]
Accipitriformes							
Accipitridae							
*Harpia harpyja*, HHA	58	5, 6, 19, 21, 24	1, 3	2p, 10, 18, 23	4, 14	2q, 20	de Oliveira et al., [19]
*Pseudastur albicollis*, PAL (= *Leucopternis albicollis*)	66	3p + q, 6, 7, 15, 18	2, 4, 20	9, 13, 17, 26	1, 16	5, 14q	de Oliveira et al., [9]
*Buteogallus meridionalis*, BME *Buteo nitidus*, BNI (= *Asturina nitida*) *Rupornis magnirostris*, RMA	68	3p + q, 6, 7, 15, 18	2, 4, 20	9, 13, 17, 26	1, 16	5, 14q	de Oliveira et al., [16]
*Gypaetus barbatus*, GBA	60	7, 8p, 11, 12q	1q, 2, 14q, 23q	8q, 13, 21q, 22q	3, 16	15q, 20	Nanda et al., [20]
*Gyps fulvus*, GFU *Gyps rueppellii*, GRU	66	7, 12, 15, 19, 20, 22	2, 3, 23	8, 16q, 21, 24	1, 13	14q, 17	Nanda et al., [20]
*Nisaetus nipalensis orientialis*, NNI	66	6, 13, 15, 19, 21, 24, 28	2, 3, 22	10, 12	1, 14	11, 16	Nishida et al., [13]
				17, 20			
*Gyps fulvus*, GFU	66	7, 12, 21, 22, 23, 26, 28	2, 3, 24	8p + q, 16q, 19, 25	1, 13	11q, 17	present study
*Gyps himalayensis*, GHI							
*Buteo buteo*, BBU	68	6q, 7, 12, 21, 22, 23, 26, 27	2, 3, 24	8p + q, 16q, 19, 25	1, 13	11q, 17	present study
Accipitriformes							
Pandionidae							
*Pandion haliaetus*, PHA	74	1q, 2q, 3p, 4q, 5p, 23, 24	9q, 17	6q, 11q, 13q, 21	4p, 8, 18	15q, 19	Nishida et al., [18]
Falconiformes							
Falconidae							
*Falco columbarius*, FCO	40	2	3q, 4q	1p, 4p	1q, 8	3p, 5q	Nishida et al., [12]
*Falco peregrinus*, FPE	50	4, 6	3, 5	7, 11	2, 13	1p, 9	Nishida et al., [12]
*Falco tinnunculus*, FTI	52	3, 5	2, 4	6, 12	1, 14	7, 10	Nishida et al., [12]

In our cladistics analysis, two species (HHA and GBA) showed clear unstable locations in the most parsimonious trees that we retrieved here (see Additional file 1: Figure S1). This was probably due to insufficient resolution of some chromosomal changes and further reciprocal painting involving HHA and GBA will be needed to clarify the relationships of these two species with Accipitridae and Pandionidae. Yet, despite HHA and GBA instability, our chromosome-based cladistic analysis retrieved several cytogenetic signatures that support some groupings within Accipitriformes (Fig. 6), with some of them having already been suggested. For instance, seven characters (3, 18, 20, 21, 27 and 28) united the PHA + all accipitrid species (i.e., Pandionidae and Accipitridae, here) in one monophyletic assemblage; three characters (19, 30 and 32) supported the PHA + GFU (hence GRU, GHI) + NNI + BBU clade; one synapomorphy (character 49) linked Neotropical buteoninae species. The three species of Falconiformes shared eight common characters (6–12 and 54), thus leading to karyotypes that differ greatly from those found in Accipitriformes. Two characters (2 and 4, i.e., the syntenic disruptions of 2p/2q and 5a/5b) seemed to link Accipitriformes and Falconiformes. However, the syntenic disruptions of 2p/2q and 5a/5b (characters 2 and 4) were also found in some Galliformes (different from the chicken) [35] and Strigiformes species [8, 23, 36]. So further analysis is needed to clarify if these characters will be convergent events or not. In addition, species in Cathartidae (the New World vultures) have typical avian karyotypes, and showed a high degree of conservation in chromosomal synteny with GGA, thus differing from other species in Accipitriformes and species in Falconiformes, including those studied here.

Besides our cladistic analysis, we also mapped the chromosomal rearrangements onto a consensus molecular tree (Additional file 2: Figure S2). Although the 50 % majority-rule tree of the diurnal birds of prey that we retrieved using chromosomal rearrangements (Fig. 6) had some differences from the molecular phylogeny (Additional file 2: Figure S2), both analyses (i.e. the cladistic analysis and mapping rearrangements onto an independently obtained topology) revealed a number of potential cytogenetic signatures for some clades investigated here. For instance, both analyses suggested that (1) falconids (Falconiformes) had unique chromosomal rearrangements, differing from that of Accipitriformes species; (2) PHA (the osprey, Pandionidae) shared several common chromosomal rearrangements with other Accipitridae species; (3) the Old World vulture of 2n = 60 (GBA) had some distinct chromosomal characters differing from those 2n = 66 Old World vultures, although the number of common characters was different in both analyses. Some common chromosomal rearrangements, such as characters 19, 30, 32, 33, were mapped onto different clades, i.e. PHA, some of Old World vultures (GFU, GHI, GRU), NNI and BBU. However, in our cladistic analysis, characters 19, 30, 32 supported these species in one assemblage. For the placement of Pandionidae (the osprey), most molecular phylogenetic studies indicate that Sagittariidae, Pandionidae and Accipitridae form a clade, with Pandionidae being more closely related to Accipitridae [26, 27, 29, 32], but one molecular phylogenetic study supported that the osprey lies within the Accipitridae [30]. The results of our current cladistic analysis provided three cytogenetic signatures (i.e. characters 19, 30 and 32) that support the latter study, and also suggest that PHA (the osprey, Pandionidae) may well be a member of Accipitridae. In addition, our cladistic results confidently indicated that BBU, a supposed buteoninae species from Palearctic, was much closer to other accipitrids (here represented by Old World vultures and NNI) than to the Neotropical buteoninae species.

To date, the reason and mechanism explaining why extensive chromosomal fissions and fusions have occurred only in Accipitriformes and Falconiformes species remains unclear. The possible origin of the peculiar characteristics of the Accipitriformes karyotypes was briefly discussed by De Boer [3]. A translocation model of the evolution of karyotype organization in Accipitridae was also proposed based on computer simulations [37]. However, this model had to be verified by analysis of DNA sequences and localization of transposable elements in Accipitridae species [37]. Further high-resolution mapping and sequence analysis of evolutionary synteny breakpoints are required to elucidate the molecular mechanisms that underlie such extensive chromosomal rearrangements. In addition, the species in Sagittariidae and the two accipitrid species with diploid number differing from that of the majority of accipitrids (2n = 66–68), northern goshawk (*Accipiter gentilis*) with 2n = 78 and crested eagle (*Morphnus guianensis*) with 2n = 54, remain to be studied by comparative chromosome painting. The chromosome painting data in these species may be the key to understand the whole karyotype evolution in Accipitriformes.

## Conclusion

We here established comparative chromosome maps between BOE and the 2n = 66 Old World vultures (GFU and GHI) and the 2n = 68 BBU by cross-species chromosome painting using two sets of probes from avian species with atypical karyotypes (BOE and GFU). Our results indicated that at least eleven syntenic disruptions and eight segmental associations were shared by the 2n = 66 Old World vultures and the 2n = 68 BBU, thus indicating that BBU could be more closely-related to other accipitrids than to the Neotropical buteoninae species (PAL, BNI, BME and RMI). Our investigations within the diurnal birds of prey have revealed some cytogenetic signatures for different lineages and provided support for the groupings of (i) Falconiformes, and (ii) Accipitridae + Pandionidae. Finally, our chromosome-based cladistic analysis reinforced the proposition that the osprey (PHA) might be a member of Accipitridae.

## Methods

### Cell culture, metaphase preparations and chromosome nomenclature

Three accipitrid species belonging to two different genera, namely the griffon vulture (*G. fulvus*, GFU), Himalayan griffon (*G. himalayensis*, GHI) and common buzzard (*B. buteo*, BBU), and one Charadriiformes species, the stone curlew (*B. oedicnemus*, BOE) were investigated here. The BOE and GFU fibroblast cell lines were obtained from the Paris Natural History Museum and the GHI fibroblast cell line (KCB200021) was provided by Kunming Cell Bank, the Chinese Academy of Sciences. Mitotic chromosomes of BBU were obtained from a BBU chromosomal suspension stored in Kunming Cell Bank, the Chinese Academy of Sciences. We did not undertake any animal research that would require ethics approval to obtain the samples.

Cell culture, metaphase preparation and slide preparation were carried out following conventional methods [14]. The chromosomes of GFU and BOE were numbered according to previously published karyotypes [3, 14]. The karyotype of GHI was arranged according to that of GFU. To facilitate inter-specific comparison of the homologous chromosome segments within Accipitridae, the DAPI-banded BBU karyotype was arranged according to GFU and GHI karyotypes, rather than to the karyotype reported previously [5].

### Flow sorting and generation of chromosome-specific painting probes

Chromosome preparations of GFU for flow sorting followed the method described previously [38, 39], and were stained with chromomycin A3 (40 μg/ml, Sigma) and Hoechst 33258 (2 μg/ml, Sigma). Sorting was carried out using a dual-laser cell sorter (FACStar Plus, Becton Dickinson). Chromosome-specific paints for GFU were generated from flow-sorted chromosomes by degenerate oligonucleotide-primed polymerase chain reaction (DOP-PCR) [39]. DOP-PCR amplified chromosome-specific DNAs were labeled with biotin-16-dUTP, FITC-12-dUTP (Roche) or Cy3-dUTP (Amersham) during secondary DOP-PCR amplification. The set of BOE painting probes that was used in this study was generated previously from flow-sorted chromosomes [14].

### FISH, image capture and processing

Cross-species chromosome painting, image capture and processing were carried out following Nie et al. 2009 [14]. Hybridization signals were assigned to specific chromosomes or chromosome regions defined by inverted DAPI (i.e. 4′, 6-diamidino-2-phenylindole)-banding patterns.

### Chromosomal character matrix

To date, including the current study, chromosome painting has been performed in seventeen species belonging to four families of the diurnal birds of prey using one of four sets of chromosome painting probes developed from the following species: the chicken (*G. gallus*, GGA), the white hawk (*F. albicollis*, PAL), the stone curlew (*B. oedicnemus*, BOE) and the griffon vulture (*G. fulvus*, GFU). The establishment of chromosomal homologies between GGA and PAL, GGA and BOE, and between BOE and GFU allowed us to integrate all chromosomal homologies that were already available in the diurnal birds of prey using GGA as a reference. Unlike in other avian species, the syntenies of GGA 1, 2 and 3 were each broken into two pieces in Falconidae, and more than three pieces in Accipitridae and Pandionidae. Based on the reciprocal chromosome painting data between BOE and GGA, PAL and GGA, and BOE and GFU as well as chromosome banding comparison among homologues that correspond to subchromosomal regions on GGA chromosomes 1–3, most of the syntenic breakpoints on GGA 1, 2 and 3 seem to be identical in Accipitridae and Pandionidae. Only a couple of further syntenic disruptions in one or two GGA 1 subchromosomal segments resulted in the difference in the numbers of homologous GGA 1 segments among different species. Due to the lack of reverse painting data for most studied Falconiformes and Accipitriformes species and the lack of single GGA microchromosome probes, we could not determine if all chromosomal rearrangements revealed by GGA probes were of the same origin. For such chromosomal rearrangements that we could not define their origins, we treated them as different characters. Table 1 lists the chromosomal rearrangement characters for seventeen studied Falconiformes and Accipitriformes species.

### Cladistic analysis and character mapping

A cladistic analysis using 59 chromosomal characters (Table 1) was conducted as recommended by Dobigny et al. (2004) [40]. Briefly, it consisted in scoring syntenic disruptions or associations as unordered and equally weighted characters, and their presence/absence as character states. *Gallus gallus* (GGA, Galliformes) was used as the only outgroup. The most parsimonious topologies were investigated through an exhaustive search (initial MaxTrees = 100; MulTree option) conducted under PAUP 4.0 b [41]. The character changes were polarized *a posteriori* using the outgroup comparison criterion [42].

In parallel to the cladistic analysis, the chromosomal differences inferred by ZooFISH were also mapped onto an independently obtained phylogenetic tree. Since no phylogenetic study including all the species studied in our own present work has ever been conducted, we explored the literature and built a consensus topology on the basis of recently published molecular phylogenetic trees [29, 30, 33]. We took this latter topology as the reference species tree, and mapped the 59 chromosomal changes onto it.

## Additional files

Additional file 1: Figure S1. The 10 equally and most parsimonious trees. (DOC 63 kb) Additional file 2: Figure S2. Mapping of all the 59 chromosomal differences inferred by Zoo-FISH onto a consensus molecular phylogenetic tree. Since no existing molecular phylogeny includes all or most of the species that we studied in this paper, we had to rely on the literature to reconstruct a combined consensus tree based on several publications (for the relationships of species studied here in Accipitriformes, we combined the trees in Griffiths et al. [29] and Barrowclough et al. [32]; for the relationships between Falconiforms and Accipitriformes, and between Cathartidae and other species in Accipitriformes, Jarvis et al. (2014) tree was used). * indicates apparently homoplasic (i.e., convergent or reversal) characters. (JPEG 560 kb)

## Abbreviations

BBU: *Buteo buteo*
BNI: *Buteo nitida* (= *Asturina nitida*)
BOE: *Burhinus oedicnemus*
BME: *Buteogallus meridionalis*
CAU: *Cathartes aura*
DAPI: 4′, 6-diamidino-2-phenylindole
DOP-PCR: Degenerate oligonucleotide-primed PCR
FCO: *Falco columbarius*
FISH: Fluorescent *in situ* hybridization
FPE: *Falco peregrinus*
FTI: *Falco tinnunculus*
GBA: *Gypaetus barbatus*
GCA: *Gymnogyps californianus*
GFU: *Gyps fulvus*
GGA: *Gallus gallus*
GHI: *Gyps himalayensis*
GRU: *Gyps rueppellii*
HHA: *Harpia harpyja*
NNI: *Nisaetus nipalensis orientialis*
PAL: *Pseudastur albicollis* (= *Leucopternis albicollis*)
PHA: *Pandion haliaetus*
RMA: *Rupornis magnirostris*
