# Supplementary material for: Multidirectional chromosome painting substantiates the occurrence of extensive genomic reshuffling within Accipitriformes
Source: BMC Evol Biol. 2015 Sep 26;15:205. doi: 10.1186/s12862-015-0484-0 (PMC4583764; doi:10.1186/s12862-015-0484-0)
Supplement: Additional file 1: Figure S1. — The 10 equally and most parsimonious trees. (DOC 63 kb) [file 12862_2015_484_MOESM1_ESM.doc]

**Strict consensus of the 10 most parsimonious trees**

/-------------------------------------------------------------------------- GGA

|

|

/---------------------------------------------------------------------- GCA

|

| /------------------------------------------------- FTI

| |

+----------------+ /------------------------- FPE

| \---------------+

| \------------------------- FCO

|

| /------------------------------------------------- PHA

| |

| +------------------------------------------------- GBA

| |

| +------------------------------------------------- GFU

| |

| +------------------------------------------------- HHA

\-----------------+

+------------------------------------------------- NNI

|

| /------------------------- ANI

+---------------+

| \------------------------- LAL

|

\------------------------------------------------- BBU

**50% majority-rule of the 10 most parsimonious trees**

/---------------------------------------------------------------------- GGA(1)

|

|

/---------------------------------------------------------------- GCA(13)

|

| /----------------------------------- FTI(2)

| |

+-----100---------------+ /------------------ FPE(3)

| \---100----+

| \------------------ FCO(4)

|

| /------------------ PHA(5)

| |

| +------------------ GFU(7)

| /---90----+

| | +------------------ NNI(9)

| | |

| /----70----+ \------------------ BBU(12)

| | |

| | \----------------------------------- GBA(6)

| |

\-100-----+----------------------------------------------------- HHA(8)

|

| /------------------ ANI(10)

\----100--------------+

\------------------ LAL(11)

**The 10 equally and most parsimonious trees**

**Tree number 1 (rooted using user-specified outgroup)**

/-------------------------------------------------------------------------- GGA

|

|

/----------------------------------------------------------------- GCA

|

| /------------------------- FTI

| |

+-------------------------------------+ /------------ FPE

| \------------+

| \------------ FCO

|

| /------------------------------------- PHA

| |

| | /------------ GBA

| /---------+ /------------+

| | | | \------------ NNI

| | | |

| | \-----------+------------------------- GFU

| /------------+ |

| | | \------------------------- BBU

| | |

\-----------+ \------------------------------------------------- HHA

|

| /------------ ANI

\------------------------------------+

\------------ LAL

**Tree number 2 (rooted using user-specified outgroup)**

/-------------------------------------------------------------------------- GGA

|

|

/----------------------------------------------------------------- GCA

|

| /------------------------------ FTI

| |

+--------------------------------+ /--------------- FPE

| \----------+

| \--------------- FCO

|

| /--------------- PHA

| |

| +--------------- GFU

| /-----------+

| | +--------------- NNI

| | |

| /----------+ \--------------- BBU

| | |

| /----------+ \------------------------------ GBA

| | |

| | \-------------------------------------------- HHA

\--------------+

| /--------------- ANI

\--------------------------------+

\--------------- LAL

**Tree number 3 (rooted using user-specified outgroup)**

/-------------------------------------------------------------------------- GGA

|

|

/----------------------------------------------------------------- GCA

|

| /------------------------- FTI

| |

+------------------------------------+ /------------ FPE

| \---------+

| \------------ FCO

|

| /------------ PHA

| |

| /---------+------------ GFU

| | |

| /--------+ \------------ BBU

| | |

| /---------+ \------------------------- NNI

| | |

| /---------+ \------------------------------------- GBA

| | |

| | \------------------------------------------------- HHA

\---------+

| /------------ ANI

\------------------------------------+

\------------ LAL

**Tree number 4 (rooted using user-specified outgroup)**

/-------------------------------------------------------------------------- GGA

|

|

/----------------------------------------------------------------- GCA

|

| /------------------------------ FTI

| |

+--------------------------------+ /--------------- FPE

| \----------+

| \--------------- FCO

|

| /--------------- PHA

| |

| +--------------- GFU

| /----------+

| | +--------------- NNI

| | |

| /----------+ \--------------- BBU

| | |

| | \------------------------------ GBA

| /--------------+

| | | /--------------- ANI

| | \---------------------+

\-----------+ \--------------- LAL

|

\----------------------------------------------------------- HHA

**Tree number 5 (rooted using user-specified outgroup)**

/-------------------------------------------------------------------------- GGA

|

|

/----------------------------------------------------------------- GCA

|

| /------------------------- FTI

| |

+------------------------------------------------+ /------------ FPE

| \------------+

| \------------ FCO

|

| /------------ PHA

| |

| /------------+------------ GFU

| | |

| /-----------+ \------------ BBU

| | |

| /-----------+ \------------------------- NNI

| | |

| | \------------------------------------- GBA

| /------------+

| | | /------------ ANI

| | \------------------------------------+

\-----------+ \------------ LAL

|

\-------------------------------------------------------------- HHA

**Tree number 6 (rooted using user-specified outgroup)**

/-------------------------------------------------------------------------- GGA

|

|

/----------------------------------------------------------------- GCA

|

| /------------------------- FTI

| |

+-------------------------------------+ /------------ FPE

| \----------+

| \------------ FCO

|

| /------------------------- PHA

| |

| /--------+ /------------ GFU

| | | |

| | \----------+------------ NNI

| /--------+ |

| | | \------------ BBU

| | |

| /---------+ \------------------------------------- GBA

| | |

| | \------------------------------------------------- HHA

\---------+

| /------------ ANI

\------------------------------------+

\------------ LAL

**Tree number 7 (rooted using user-specified outgroup)**

/-------------------------------------------------------------------------- GGA

|

|

/----------------------------------------------------------------- GCA

|

| /------------------------- FTI

| |

+-------------------------------------+ /------------ FPE

| \-------+

| \------------ FCO

|

| /------------------------- PHA

| |

| /--------+ /------------ GFU

| | | |

| | \----------+------------ NNI

| /--------+ |

| | | \------------ BBU

| | |

| /---------+ \------------------------------------- GBA

| | |

| | | /------------ ANI

\---------+ \--------------------------+

| \------------ LAL

|

\-------------------------------------------------------------- HHA

**Tree number 8 (rooted using user-specified outgroup)**

/-------------------------------------------------------------------------- GGA

|

|

/----------------------------------------------------------------- GCA

|

| /------------------------- FTI

| |

+-------------------------------------+ /------------ FPE

| \-----------+

| \------------ FCO

|

| /------------------------- PHA

| |

| /--------+ /------------ GFU

| | | |

| | \----------+------------ NNI

| /--------+ |

| | | \------------ BBU

| | |

| /--------+ \------------------------------------- HHA

| | |

| | \------------------------------------------------- GBA

\---------+

| /------------ ANI

\-------------------------------------+

\------------ LAL

**Tree number 9 (rooted using user-specified outgroup)**

/-------------------------------------------------------------------------- GGA

|

|

/----------------------------------------------------------------- GCA

|

| /------------------------------ FTI

| |

+---------------------------------+ /--------------- FPE

| \-----------+

| \--------------- FCO

|

| /------------------------------ PHA

| |

| /----------+ /--------------- GFU

| | | |

| | \-----------+--------------- NNI

| /----------+ |

| | | \--------------- BBU

| | |

| | \----------------------------------------- HHA

\-----------+

| /------------------------------ GBA

| |

\----------------------+ /--------------- ANI

\---------+

\--------------- LAL

**Tree number 10 (rooted using user-specified outgroup)**

/-------------------------------------------------------------------------- GGA

|

|

/----------------------------------------------------------------- GCA

|

| /------------------------- FTI

| |

+-------------------------------------+ /------------ FPE

| \----------+

| \------------ FCO

|

| /------------------------- PHA

| |

| /--------+ /------------ GFU

| | | |

| | \----------+------------ NNI

| /---------+ |

| | | \------------ BBU

| | |

| /---------+ \------------------------------------- HHA

| | |

| | | /------------ ANI

\---------+ \------------------------+

| \------------ LAL

|

\-------------------------------------------------------------- GBA
