# Supplementary figures and images for: Multidirectional chromosome painting substantiates the occurrence of extensive genomic reshuffling within Accipitriformes
Source: BMC Evol Biol. 2015 Sep 26;15:205. doi: 10.1186/s12862-015-0484-0 (PMC4583764; doi:10.1186/s12862-015-0484-0)

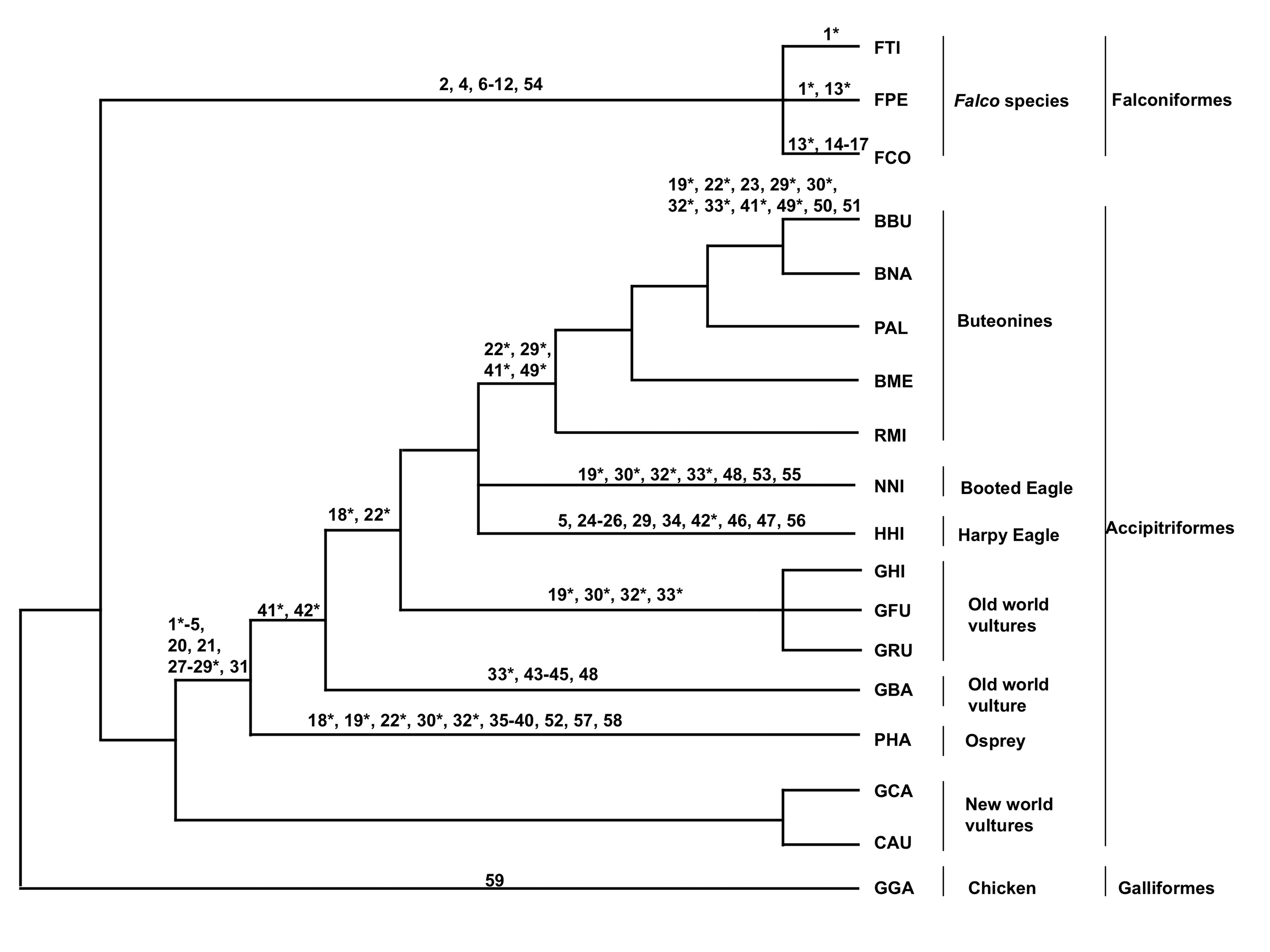

Supplement: Additional file 2: Figure S2. — Mapping of all the 59 chromosomal differences inferred by Zoo-FISH onto a consensus molecular phylogenetic tree. Since no existing molecular phylogeny includes all or most of the species that we studied in this paper, we had to rely on the literature to reconstruct a combined consensus tree based on several publications (for the relationships of species studied here in Accipitriformes, we combined the trees in Griffiths et al. [29] and Barrowclough et al. [32]; for the relationships between Falconiforms and Accipitriformes, and between Cathartidae and other species in Accipitriformes, Jarvis et al. (2014) tree was used). * indicates apparently homoplasic (i.e., convergent or reversal) characters. (JPEG 560 kb) [file 12862_2015_484_MOESM2_ESM.jpg]
